# Toxic Absence: Why Leader Presence Matters in Times of Crisis

**DOI:** 10.1155/2023/1315904

**Published:** 2023-11-07

**Authors:** Robert McMurray, Nicki Credland, Martyn Griffin, Peter Hamilton, Oonagh Harness

**Affiliations:** ^1^University of Sheffield, Sheffield, UK; ^2^University of Hull, Hull, UK; ^3^Durham University, Durham, UK; ^4^Northumbria University, Newcastle upon Tyne, UK

## Abstract

**Aims:**

This study examines the importance of senior-leader presence on the “frontline” in times of crisis.

**Background:**

The COVID-19 pandemic placed unprecedented demands on nurses charged with delivering critical care. Extant research suggests that the active presence of ward-level leaders has an important role to play in supporting frontline staff and mediating the negative impacts of stress and burnout. There is little evidence on the impact of senior leader presence or absence on the experience of frontline critical care nurses, particularly at times of crisis.

**Methods:**

A three-phase qualitative interview study of critical care nurses in the UK and Ireland. A total of 107 semistructured interviews with 54 nurses representing 38 different healthcare units.

**Results:**

Senior-leader presence at the time of crisis serves as an important symbol of organisational support. Where senior leaders are not meaningfully present, they risk allowing the necessary pain of difficult work situations to become toxic. Toxicity is manifested with increased staff stress, emotional ills, absence, and turnover.

**Conclusions:**

Senior leaders must balance their responsibilities for strategy and structures with the frontline presence required to shape a positive emotional climate. *Implications for Nursing Management*. Senior managers should consider supplementing their strategic focus with punctuated returns to the floor. Symbolically, leaders who get their hands dirty embody a sense of mutual struggle and practical support. Managerially, time on the floor increases the opportunities for collecting primary data to improve decision-making and support.

## 1. Introduction

Presence is an important component of nursing care [1, 2]. It describes an immersive interpersonal engagement with the patient as a person, tending to their specific claims, concerns, and needs as part of high-quality care. There is a small but growing body of evidence to suggest that presence might also be a central component in the support of nurses themselves. Ward-leader presence can help support and develop nurses in their work and careers [3, 4]. What is less well understood is the role “senior-leader” presence has on nursing work and well-being, particularly during times of crisis.

### 1.1. Critical Care, Presence, and Organisational Pain

At the best of times, critical care nursing is characterised by a “predictably unpredictable environment of chaos” [5]. The pandemic only heightened this sense with major changes in structures, routines, processes, location, staff, disease mix, and work environment. As infections, admissions, and deaths rose, critical care nurses fought to save patients who were sedated, intubated, often ventilated, and regularly isolated from those they loved [6]. Through it all, frontline nurses continued to provide much-needed human presence, where presence is about being there, face-to-face with the patient, and accepting responsibility for a unique individual who should never be reduced to a disease type or patient category [1].

Presence is linked to, but goes beyond, daily routines, technical competencies, and the task-based concerns that characterise so much nursing work [7]. It speaks to a holistic, nontechnical practice that fundamentally shapes patient experiences of care and is associated with the alleviation of suffering and distress, reduced isolation, empowerment, enhanced cooperation, and improved outcomes [1, 7, 8]. As such, presence is a central tenet of compassionate, high-quality nursing care [8–10].

The presence at the bedside of the patient took a painful toll during the pandemic for nurses encumbered by PPE [11] and traumatised by the effects of COVID-19 working [12]. A 2021 UK survey found substantial rates of probable mental health disorders and thoughts of self-harm amongst ICU staff' with “nearly one in five nurses…working in ICU report(ing) thoughts of self-harm or suicide” [12]. These difficulties were “especially prevalent in nurses” [12], who were substantially more likely to suffer serious mental health problems during the pandemic compared to other healthcare occupations. High levels of anxiety, depression, and PTSD in nurses on the frontline have also been found in similar studies in China [13], Italy [14] France [15], Turkey [16], and Canada [17]. In the USA, 66% of critical care nurses surveyed considered leaving nursing in light of pandemic experiences [18]. In organisational terms, these are the toxic effects of painful work.

### 1.2. Toxicity, Presence, and Leaders

Frost [19] notes that pain is a fact of organisational life. People make unreasonable demands, communication goes badly, coworkers are insensitive, and leaders may be hurtful. Work can also be excessively demanding–especially at times of crisis [20]. These painful experiences become toxic when they are left unrecognised and untreated, particularly in the face of “emotionally insensitive attitudes and actions on the part of managers” [19]. Untreated, everyday organisational pain can poison “a person or an entire system: toxins spread and seep, often undetected” [19]. As in the case of critical care nursing during the pandemic, these toxins manifest as emotional distress, absence, and turnover on the part of workers [12, 18]. And yet, toxicity can be ameliorated [21].

Frost [19] contends that leaders have a significant role in tackling the sources and effects of toxicity. He specifically notes the importance of leaders “maintaining a presence in the face of great suffering” [19], where presence infers person-centredness, compassion, active listening, and acknowledging and responding to the needs of others (note the overlaps between the prescription for leader presence at the site of toxicity and nurse presence at the bedside of the patient [1, 3, 4, 7, 8, 19]). Indeed, within the context of ward-level management, leader presence is positively associated with nurse development, better communication, and improved formal and informal support [3, 22, 23]. Beyond healthcare, there is a growing body of research that recognises the importance of dealing with the sources of pain identified by Frost [19], whether this is through the presence of HR departments in handling pain [24], boards of directors guarding against toxic cultures [25], managers promoting ethics of care [26, 27], or reflective leaders pursing compassionate rather than harmful decisions and behaviours [20, 28, 29]. Within the context of COVID-19, there has also been a growing emphasis on the need for leaders to demonstrate a commitment to evidence-based decisions, effective communication, shared purpose, empathy, well-being, and trust [20, 30–33].

What is not clear—particularly in respect of critical care nursing—is the role that meaningful *senior-leader presence* may have in tackling organisational pain and preventing toxicity at times of crisis. For the purposes of this study, senior leaders are identified as nurses in band 7 or above. Often referred to as “Senior Sisters” within the UK NHS, these nurses take on management responsibilities, have highly specialised knowledge (often at Masters level), and may undertake tasks usually associated with doctors. Senior leaders also include medical consultants and nonclinical leaders above ward level (e.g., CEOs, heads of human resources). These categorisations are also reflected in the data (below) where nurses at level 7 or above are referred to as “seniors” by respondents.

Focusing on these more senior leaders is important given that extant studies tend to be small-scale and focused on ward-level leadership [3, 4]. Where research does talk about the role of more senior leaders (see [22], it has little to say about either the presence or absence during times of crisis. Analysing the interview data from 54 critical care nurses in the UK and Ireland, we consider how nurses perceived the presence and absence of their senior leaders on the frontline during the first two waves of the COVID-19 pandemic. We assess the conditions in which critical care nurses worked, the impact of those conditions on their well-being, and the extent to which senior-leader presence and absence ameliorated or exacerbated those conditions.

## 2. Methods

### 2.1. Sample

A study of critical care nurses was undertaken so that we might better understand the work and experiences of those working at the extreme edge [34, 35] of the COVID-19 pandemic: work rendered extreme by virtue of proximity to death, personal physical danger, and gruelling working conditions. Participants were targeted through a call on a social media platform by the British Association of Critical Care Nurses (BACCN). A total of 54 nurses were recruited from 38 hospitals in the UK (*n* = 52) and Ireland (*n* = 2). All participants were critical care nurses, with between 2 and over 30 years' experience, who worked in an adult intensive care unit during the COVID-19 pandemic.

### 2.2. Data Collection and Instruments

The data were gathered through longitudinal, semistructured interviews. Three phases of interviews were conducted with a view to considering whether particular issues, concerns, or themes persisted, diminished, or emerged as the crisis unfolded (e.g., did pandemic working have a cumulative impact on nurses; did support improve over time?). The first phase of interviewing commenced in September/October 2020, the second in January/February 2021, and the final phase in May/September 2021. The timing of the interviews was both to accommodate participants' availability but also to capture “critical moments of change and transitions” [36] throughout the pandemic.  Table 1 presents the attrition rates and average duration of interviews across the three phases (reflecting increasingly restricted availability due to work and ill-health).

Interviews were conducted using video conferencing software (e.g., Zoom, Teams, Facetime) to comply with the social distancing requirements and enable swift access to participants nationally. Semistructured interview guides identified key issues for discussion (career, experience, COVID-19, support, emotions, challenges, and changes) while retaining flexibility to consider emergent issues and concerns. Conscious of the potentially emotional nature of interviews, we provided a direct referral pathway to free counselling through BACCN for participants.

### 2.3. Data Analysis

The interviews were professionally transcribed. The data were analysed through NVivo using an inductive thematic approach based on a constant comparative method [37] with a view to examining and comparing the actions, experiences, processes, reactions, and interpretations of those engaged in critical care COVID-19 work. Detailed reading and coding of the data by the first and third authors individually was followed by careful comparison of emergent codes, themes, and patterns before reanalysis. This iterative process resulted in eleven primary themes (see Table 2) that were then collated under three meta-headings. Within the analysis (below), quotes are used to represent a wider class of issues/codes, with longer or multiple quotes being used to build a narrative sense of the category, issues, and lived experience.

## 3. Results

### 3.1. The Organisational Pain of Crisis

All of the nurses interviewed experienced a sense of personal and professional upheaval in the face of the pandemic. The scale of demand threatened to overwhelm critical care units. Nurses were required to transition from dealing with one or two patients in the ICU to caring for up to six critically ill COVID-19 patients at a time. Treatment plans were unclear, and the initial prognosis was bleak for prevaccine patients. Normal modes of working were undone. New protocols were devised, implemented, and changed to cope with the emergent disease and develop knowledge. Uncomfortable and depersonalising PPE became mandatory (though not always available during the first wave). Wards were isolated, expanded, or moved. Noncritical care specialists drafted from other wards had to be trained and overseen. Death tolls rose, and families were excluded. The impact on critical care nurses was immediate, enduring, and profound.

“I'm trying not to use swear words here. It was shit. It was awful”. (CCN20-phase1)

Bearing witness to large-scale deathscapes, patient suffering, and family trauma took an emotional toll on nurses. Some spoke of being “in counselling now” (CCN29-phase1) while for others the strain of long shifts, inadequate provision, and patient suffering led to hysteria.

“I went for a run to the park… I thought I'd lost my mind. I had to stop running and I was just hysterical crying in the middle of a park and I couldn't calm it down… [Next] morning, from the moment I opened my eyes, hysterical, again” (CNN42-phase1).

In each phase of interviewing nurses spoke of how the burden of dealing with difficult emotions during successive COVID-19 waves led to emotional pain and psychological suffering. Examples included “night terrors” (CCN44-phase1) sectioning and suicidal thoughts:

“… more nightmares and like flashbacks about my patients; and yeah–this is the bit that will like shock you now–I was sectioned twice over the summer under Section 2” (CNN16-phase1)

“I had a really, really down month where I was feeling really suicidal and all I could think in my head was I just want to go into work and get some drugs and just kill myself” (CNN16-phase3).

In some hospitals' sickness, absenteeism, and nurse turnover led to “a really bad staffing crisis” because “everyone's dropping like flies” (CNN2-phase2) as the toxic effects of COVID-19 working manifested through “a lot of nurses off sick with stress, anxiety, and posttraumatic stress disorder” (CNN48-phase2). Faced with these painful experiences, nurses spoke of the need for support, coupled with frustration where it was absent.

### 3.2. Toxic Absence

Nurses spoke highly of informal peer support on the wards (among band 5s and band 6s) though this was limited by the closure of staff rooms, lack of time, and difficulties communicating through the strictures of PPE and social distancing. There was also gratitude for what Søvold et al. [38] label “short-term mood boosters” such as free food, pampering, and clapping. Counselling or debriefing after difficult shifts was wanted but was all too often unavailable or inaccessible.

Immediate collegiate support was contrasted with what many described as the failures of hospital leadership and management. There was a perception that leaders (from band 7 upward) neither understood nor supported nurses on the ground.

“The only people that has supported us is each other… I don't think our management have got any real understanding of what the intensive care nurses have been through, and I don't feel like we've been offered any real support really…. We're all really scared at the minute” (CNN23-phase1)

Many felt abandoned by senior leaders who appear to have “hid in their office.”

“It's almost like they [senior-leaders] hid in the office. … It's almost like you were PPE'd up and they pushed you in and shut the doors, and then they're like, “You've got to stay in there now.” It's like, “Are you joking?” (laughs). Completely abandoned. Honestly, that's the only word I can describe it with, abandoned by the senior team. It was awful.” (CNN26-phase1)

Others referred to nurses being used as “cannon fodder” (CNN012-phase1) by senior leaders who would rather stay in their offices than share the risks that nurses faced hourly.

“When we were working shifts, gruelling shifts–we'd have seniors sort of passing messages through the door because they didn't want to come in; and just sort of opening the door and shouting things; “this has to be done.” (CNN045-phase1)

“It's an ongoing joke in my work that we never saw any of our band 7s in the pandemic and we were like, maybe they were on holiday because we–I never saw one of my band 7s in PPE once! It just didn't happen.” (CCN22-phase1)

Similar criticism was levelled at leaders from other professions. Doctors in particular were singled out. Compared to nurses as “cannon fodder,” doctors were positioned as too important to be risked on COVID-19 wards.

“There was definitely that sort of hierarchical, “we're doctors, we're important, if we get it, then who's going to look after the patients.” We were like, you're not even looking after the fucking patients, it's us that's looking after the patients.” (CNN12-phase1)

“What you found as well is that none of the doctors, not even the consultants, nobody liked being on the unit. You felt really alone. Really unsupported and in a dangerous environment if I'm perfectly honest. We were really isolated.” (CCN23-phase1)

“And a lot of us did say that the doctors, that there should be more–there should have been more of a presence.” (CCN41-phase1)

For some, the absence of senior leaders stood as a repudiation of the suggestion that nurses and their leaders were “all in it together.”

“They tell us “we understand. Our door's always open. Come and talk to us. We're all in this together.” More and more of us are thinking well, “no we're not, are we? We're not in this together. You have never been on the unit in full PPE for hours on end. You've never dealt with the relatives. You've never dealt with the distressed patients.” We really feel like we're just sort of, I don't know, almost pawns that are just being sent down the pit, if you like.” (CCN47-phase1)

Symbolically, the absence of senior leaders fed a discourse of abandonment. That leaders appeared unwilling to serve alongside critical care nurses on the frontline fed a narrative of neglect, disillusionment, and ultimately demotivation. The apparent desire to protect seniors and other occupational groups reinforced a sense of nurses as low-value players “pawns” readily sacrificed at the start of a longer campaign by leaders who “don't really care about how you are doing; how hard it was for you” (CNN22-phase1). For nurses, it spoke to a lack of understanding rooted in detachment.

### 3.3. Detached Leadership

For nurses, absent leaders lacked the on-the-ground knowledge and understanding required to “give any support” (CNN20-phase2) to the frontline:

“So the management never came in. Not once did they walk through the door. So, they couldn't even see the chaos of stock and patients and all this kind of stuff. Then every now and again you'd get a little note saying “Please ensure the mouth care is done on these patients” and you'd think, sod off (laughs)!” (CCN37-phase1)

In the above extract, lack of meaningful presence is linked to organisational failings associated with “chaos” and out-of-touch leaders whose requests engender resistance. The perceived detachment of senior leaders from the lived experience of COVID-19 work meant that top-down decisions were seen by nurses as misplaced, unhelpful, or unrealistic. There was disillusionment and anger at being criticised for not having completed business-as-usual organisational tasks such as performance reviews.

“when the second wave was over, within the first week … I had an e-mail saying, “You've got eight PDRs that are all out of date. When are you getting the—?” It's like, right, that's over with now, you crack on and get back (laughs) to your normal job (CCN21-phase2).

By phase three, the result was “a lot of bad feelings towards managers who are just shovelling more work your way and not supporting you” (CCN33-phase3). What nurses wanted was meaningful leader presence. They wanted a physical, temporal, and relational commitment to being there, helping, understanding, and caring for those who risked and laboured at the frontline. Cursory or “other” focused leader appearances that were “just for show” (CNN33-phase1) did more harm than good.

“I think one of the band sevens, I think I saw her on there once, and that was because she was showing the chief exec around. It caused a bit of bitterness really” (CNN47-Phase1)

‘As soon as a camera comes around to report, they're [doctors] in PPE and they're in there and they're pretending that they're always there.” (CCN25-phase2)

“watching the sevens parade around in a yellow apron and an FFP3 but not actually interact with us, it kind of stung …. and you could hear people just having a bit of a weep” (CCN6-phase1).

The above examples of leader presence for “show” served to increase toxicity by invoking contempt, bitterness, and tears. Nurses wanted leaders who could make decisions based on visibility and first-hand understanding of conditions on the ground.

“I think looking back, I think having our unit manager, matron, more visible. Get your PPE on, come in and help us roll patients. Don't worry about paperwork over there, that can be done another day. I suppose it's understanding what pressures we're under… it's for senior people to take that on board and listen.” (CNN46-phase1)

“I think if they were more visible I think that would have helped… I think if you had proper leadership, it would've been so much easier” (CNN26-phase1)

Occasional examples of where leaders were meaningfully present and were seen as supportive stood in sharp relief to the experiences of absence and abandonment. As one critical care nurse noted:

“I mean some of the senior nurses are absolutely fantastic–the majority of them are…. if everyone's supporting each other, I think people feel less stressed, which you know, is definitely going to have a positive impact on everything really, you know, work, getting things done, looking after people efficiently.” (CCN9-phase1)

Where senior leaders were present and nurses felt supported, they were far more likely to report positively on their ability to work through and cope with crisis. They responded positively to senior leaders who were present to ask “how are you feeling, how are you getting through this” (CNN45-phase1) and applauded matrons whose presence meant they were able to “see what's needed” and were willing to “shout loud” in order to provide support (CCN41-phase2). Senior leader's (medical or nursing) willingness to get their hands dirty —“to clean a patient up, full of poo” (CNN46-phase1)—was also taken as a sign of togetherness. In these contexts, presence mattered.

## 4. Discussion

This paper has focused on critical care nurse accounts of senior-leader absence during a period of crisis. The crisis—the global COVID-19 pandemic—radically transformed the nature, quantity, and experience of work for those on the frontline of our critical care units. An already dynamic and complex nursing environment was further complicated by uncertainty, changing rules, excessive demands, long days, and difficult patient/family encounters that took a painful toll on nurses. As observed elsewhere, the organisational impacts included increased stress, exhaustion, mental illness, absenteeism, and exit on the part of nurses [39].

While the extant literature acknowledges the important role that leadership has to play in exacerbating or ameliorating pain and toxicity [24, 25, 28, 29] with the latter work shedding some light on the impact of the global pandemic [33, 40], there is limited understanding of the role of leadership presence/absence, particularly where people work at the edge of crisis [35]. Our research addresses this gap, extending and explaining Frost's [19] original contention that leaders should be present in the face of suffering. Specifically, we show how the absence of senior leaders from the crisis frontline exacerbated the organisational pain and personal suffering of nurses to the detriment of individuals and their organisations (with ill-health, sectioning, and professional exit being included among the more negative effects). Where senior leaders failed to maintain a “presence in the face of great suffering” [19], nurses talked of being abandoned and sacrificed by those more senior than themselves. Rather than being supported, there was a feeling that senior leaders did not care about or understand the experiences of COVID-19 nursing. Without “being-there” nurses struggled to see how senior leaders could make decisions that facilitated rather than hindered their work.

We recognise that there are good reasons why leaders might be absent: poor resourcing, time pressures, role conflicts, unrealistic expectations, and an overemphasis on surveillance metrics and measurement [3, 5, 9] which combine to relegate relational work to an afterthought. Nor are we suggesting that senior leaders should abandon their strategic and operational tasks in favour of permanent residence on the crisis frontline. What we do call for is a recognition on the part of senior leaders that their behind-the-scenes work may be undermined if they are never seen on the floor, particularly in times of crisis.

As Kanter [41] notes, senior leaders are “responsible for the big structures that serve as the cornerstones of confidence and for the human touches that shape a positive emotional climate to inspire and motivate people” [41]. This requires contextually sensitive balancing of strategic and relational responsibilities that must be finessed over time. While the “ward manager who wants to exercise nursing leadership, has to take time and be present in daily work” [3], senior-leader presence is more punctuated and adaptive depending on the issues and context (e.g., less during periods of relative stability, more at times of crisis). Such an approach responds to frontline nurse demands for senior leaders who are willing to take the time to “see things with their own eyes” [23].

Building on Frost [19], we would argue that taking time to “be there,” maintaining a meaningful presence in the face of worker suffering, is a prerequisite to providing support and preventing painful working situations from becoming toxic. While other studies emphasize the need for pandemic leaders to base decisions on evidence and work to develop a shared purpose [31], we would add that in certain contexts—such as critical care—presence on the ground is often a prerequisite to such endeavours. A degree of relational presence is part of caring and emotionally sensitive management rooted in listening and decision-making based on first-hand information [20]. It is only by visibly understanding and sensitively addressing the pain experienced by critical care nurses in times of crisis that leaders can hope to prevent difficult situations from becoming toxic.

Finally, there is the symbolic value of presence. Where leaders are seen getting their hands dirty in nursing tasks, they may reduce any sense of discriminatory hierarchy and the toxic implications of a them-and-us culture. Such presence also counters any sense of frontline workers being abandoned or sacrificed in so far as leaders can be seen to share (in some limited way) the risks and rigours faced by nurses.

### 4.1. Limitations

The study provides a timely, in-depth account of one of the most important (and extreme) care contexts during the pandemic, as experienced by those who arguably spent most time with the critically ill and dying. It reverses the tendency in management research to focus on the views and evaluations of organisational elites (though future research might compare elite and frontline perspectives). Future studies might also offer comparisons of different geographies as well as national, ethnic, and organisational cultures.

## 5. Conclusion and Implications

Our contention is not that senior leaders were the primary cause of the difficulties experienced by critical care nurses. Nor are we suggesting that leaders themselves were unaffected by the multiple demands and pain of pandemic work [11]. Our point is that senior leaders must balance their responsibilities for strategy and structures, with the frontline presence required to help address organisational pain and shape positive emotional climates [20]. Symbolically, leaders who get their hands dirty embody a sense of mutual struggle and practical support. Managerially, time on the floor increases the opportunities for collecting primary evidence on the impact the specific circumstances, actions, and resourcing with a view to improved decision-making and support.

## Figures and Tables

**Table 1:** Interview numbers and duration.

	Phase 1	Phase 2	Phase 3
Participants	54	29	24
Average interview duration (minutes)	75	55	30

**Table 2:** Analytical themes.

Primary themes	Meta-theme
Experience	Organisational pain and crisis
Pain	
Impact on nurses (burnout, exit, illness, and dismay)	

Need for support	Toxic absence
Absence	
Abandonment	
Toxicity	

Lack of understanding	Detached leadership
Demands and decisions	
Performative/show	
Desired presence/hands dirty	

## Data Availability

Informed consent was not gained to release the raw qualitative data for third party use due to ethical, confidentiality, and privacy concerns arising from the sensitive nature of the data as it pertains to individuals and their places of employment.
